# Primary care clinicians’ role in advocating for equitable healthcare access

**DOI:** 10.4102/safp.v67i1.6063

**Published:** 2025-03-03

**Authors:** Klaus B. von Pressentin, Ramprakash Kaswa, Shane Murphy, Arun Nair, Indiran Govender

**Affiliations:** 1 Department of Family, Community and Emergency Care, Faculty of Health Sciences, University of Cape Town, Cape Town, South Africa; 2 Department of Family Medicine and Rural Health, Walter Sisulu University, Mthatha, South Africa; 3 Mthatha General Hospital, Mthatha, South Africa; 4 Department of Clinical Services, Medicross, Queensland, Australia; 5 Department of Family Medicine, University of the Free State, Kimberley, South Arica; 6 Robert Mangaliso Sobukwe Hospital, Northern Cape Department of Health, Kimberley, South Arica; 7 Department of Family Medicine and Primary Health Care, School of Medicine, Sefako Makgatho Health Sciences University, Pretoria, South Africa

Advocating for equitable access to high-quality primary health care remains challenging. It requires recognising ‘access’ as a multidimensional construct. Access issues may be viewed differently from the macro-level policy perspective rather than the messy micro-level community-level realities. This editorial will explore how a macro-level approach to access issues may develop multipronged advocacy actions. Two recent examples that resonate with the Southern African healthcare community come to mind.

The first example comes from Professor Andrew Ross, the former president of the South African Academy of Family Physicians (SAAFP), who was diagnosed with myelodysplastic syndrome requiring a stem cell transplant. His experience highlights the barriers to accessing allogeneic haematopoietic stem cell transplantation (allo-HSCT). Allogeneic haematopoietic stem cell transplantation or stem cell transplantation is an increasingly used curative therapy for many life-threatening blood dyscrasias. As many patients do not have a suitable family donor, there is a critical need for matched but unrelated donors via donor registries and centres.^1^ Professor Ross publicly shared his story and launched a call for action pointing to the structural inequities disproportionately affecting certain demographic groups, especially given the urgent need to expand the global donor pool’s genetic diversity.^2^ Professor Ross partnered with the South African Bone Marrow Registry, DKMS Africa and SAAFP to spread awareness and organise community drives around South Africa.

The second example concerns the recent discontinuation of a widely used diabetic pen by a pharmaceutical company, which had a widespread, harmful impact on diabetic patients, including those in South Africa, especially in vulnerable populations such as the elderly and visually impaired persons who relied on these pens.^3^ Patients were forced to switch to older, less convenient methods, such as insulin syringes and needles. This situation highlights the vulnerability of patients who depend on consistent access to essential medications and devices and the power imbalance between pharmaceutical companies and healthcare systems. Global organisations, such as Médecins Sans Frontières, have called for more robust regulatory frameworks to prioritise patient needs and avoid double standards of care for people living with diabetes in low- and middle-income countries.^4^ The discontinuation of this diabetic pen underscores the need for advocacy to challenge corporate decisions that disproportionately affect vulnerable populations.

While these two examples – Professor Ross’s stem cell donation journey and the withdrawal of the diabetic pen – may seem unrelated, they illustrate the complex dimensions of healthcare access. Access is not simply about the availability of services. As outlined by the World Health Organization’s framework for universal health coverage (UHC), access also involves affordability, acceptability, and appropriateness.^5^ Services may be available, but remain inaccessible if they are unaffordable, culturally inappropriate, or fail to meet patients’ needs. Ethical considerations also play a significant role in the debate on healthcare access. Conflicting ethical perspectives arise when we consider healthcare from different viewpoints, such as a rights-based approach, which emphasises the individual’s right to healthcare, versus a communitarian perspective, which focuses on society’s collective responsibility to ensure equitable access. Healthcare providers must often navigate these ethical tensions by balancing the biopsychosocial considerations of their patients’ lived experiences and the realities within the health and societal structures.

**FIGURE 1:**
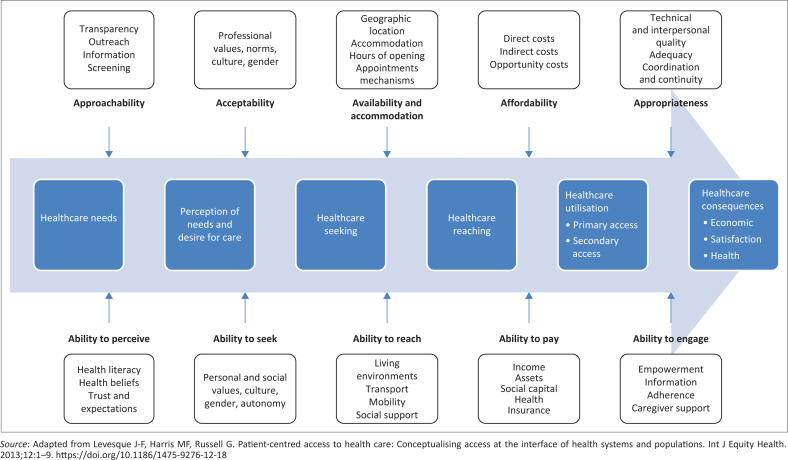
Levesque conceptual framework for healthcare access.

Levesque and colleagues proposed a helpful framework in 2013 (see Figure 1), built on previous work describing interlinked access components.^6^ The Levesque framework on patient-centred access to healthcare conceptualises access as a dynamic interaction between healthcare systems, individuals and populations involving supply-side and demand-side factors. It identifies five key dimensions of accessibility from the healthcare provider’s side (which also represents the health services’ perspective): approachability, acceptability, availability and accommodation, affordability, and appropriateness. Correspondingly, it emphasises five abilities of individuals that enable access: the ability to perceive healthcare needs, seek services, reach care, pay for care, and engage in healthcare. This comprehensive model considers the entire process, from recognising the need for care to receiving and benefiting from appropriate services. A recent scoping review, which included two South African studies, confirmed this framework’s usefulness.^5^

Understanding these dynamic interactions in the Levesque framework can help us diagnose which factors are at play in a given scenario. Despite the South African constitutional right to healthcare access, people face financial, organisational, linguistic, and cultural barriers to utilise services. For example, the discontinuation of the diabetic pen (a supply-side issue) may be addressed through more assertive advocacy that includes both healthcare providers and patients (demand-side). International bodies and collaborative platforms, such as the Global NCD Alliance, could become advocates of these patients for policy reforms that hold pharmaceutical companies accountable by engaging with funding agencies and supporting governments to scale up evidence-based interventions to meet the Sustainable Development Goals.^7^ Similarly, Professor Ross’s stem cell donation experience demonstrates the importance of building strong networks to address access barriers to underrepresented donor groups.^8^ Professional associations such as the SAAFP can play a crucial role in patient advocacy to strengthen these networks.

Advocacy for equity requires a nuanced understanding of healthcare’s complex, interrelated dimensions. Whether addressing inequities or pushing for accountability in the pharmaceutical industry, all actors within healthcare and the broader society must work together to build strong networks, including speaking out for those with silenced voices.^9^ As clinicians, we have a core sphere of influence linked to patient care, but we also have an opportunity to occupy many diverse societal roles, which may be amplified via the SAAFP network.^10^ This way, we can advocate for a more equitable healthcare system in South Africa.
